# Immune repertoire and evolutionary trajectory analysis in the development of diabetic nephropathy

**DOI:** 10.3389/fimmu.2022.1006137

**Published:** 2022-09-23

**Authors:** Zheng Ye, Yidi Zhang, Nan Huang, Shen Chen, Xiaodong Wu, Ling Li

**Affiliations:** ^1^ Department of Endocrinology, Zhongda Hospital, School of Medicine, Southeast University, Nanjing, China; ^2^ State Key Laboratory of Bioelectronics, School of Biological Science and Medical Engineering, Southeast University, Nanjing, China; ^3^ Institute of Glucose and Lipid Metabolism, Southeast University, Nanjing, China; ^4^ Department of Clinical Science and Research, Zhongda Hospital, School of Medicine, Southeast University, Nanjing, China

**Keywords:** diabetic nephropathy, immune repertoire, evolutionary trajectory, tissue microenvironment, bulk-RNAseq

## Abstract

Diabetic nephropathy (DN) is the leading cause of death and the greatest risk to the lives of people with advanced diabetes. Yet, the molecular mechanisms underlying its development and progression remain unknown. In this research, we studied the primary pathways driving DN using transcriptome sequencing and immune repertoire analysis. Firstly, we found that the diversity and abundance of the immune repertoire in late DN were significantly increased, while there was no significant change in early DN. Furthermore, B cell-mediated antibody responses may be the leading cause of DN progression. By analyzing master regulators, we found the key DN-driving transcription factors. In the late stage of DN, immune cells, fibroblasts, and epithelial cells were abundant, but other stromal cells were few. Early DN kidneys had a higher tissue stemness score than normal and advanced DN kidneys. We showed that DN progression involves proximal tubular metabolic reprogramming and stemness restoration using Monocle3. Through WGCNA, we found that co-expression modules that regulate DN progression and immune repertoire diversity mainly regulate immune-related signaling pathways. In addition, we also found that early DN had apparent activation of immune-related signaling pathways mainly enriched in immune cells. Finally, we found that activation of fibroblasts is typical of early DN. These results provide a research basis for further exploring the molecular biology and cellular mechanisms of the occurrence and development of DN and provide a theoretical basis for the prevention and treatment of DN.

## Background

Diabetic nephropathy (DN) is the most prevalent cause of renal failure (1). Although regulating blood glucose and blood pressure might lower the prevalence of DN to a certain degree, its incidence is still on the increase globally and has become a threat to public health (2, 3). Consequently, identifying the molecular processes underlying the genesis and progression of DN may aid in the identification of novel therapeutic targets to more effectively prevent and treat DN.

The development of DN may be broken down into five distinct phases: the glomerular hyperfiltration phase, the normoproteinuric period, the early DN phase, the clinical DN phase, and the renal failure phase (4). Multiple deviations from normal homeostasis, including increased systemic intra-glomerular pressure and microangiopathy triggered by haemodynamic abnormalities (hypertension), metabolic abnormalities, oxidative stress, fibrosis, and activation of the renin-angiotensin system, are now believed to be the primary mechanisms underlying the pathogenesis of DN (5, 6). Several therapies addressing these variables have been shown to significantly delay the course of DN (7, 8). Therefore, understanding the mechanism of occurrence and development of DN is of great significance for developing drugs and treatment programs for the disease.

Although much research has been done on DN, little is known about how transcriptome changes during its development (9). In a recent study comparing the transcriptomes of normal renal tissue, early DN, and late DN, genes involved in the retinoic acid pathway and the glucagon-like peptide 1 receptor were found to be protective in early DN, whereas genes associated with immune response and fibrosis might be the primary cause of DN progression (10). However, the pathogenesis of the major cell types that comprise the kidney during the progression of DN requires additional understanding. Moreover, the process of immunehistocytic alterations in renal tissues during the development of DN may play a significant role in the progression of DN since immune cells are apparent in advanced DN tissues. Even though investigations of the immune pool in IgA nephropathy have demonstrated a significant correlation between the course of renal disease and alterations in the immune repertoire (11), comparatively, few studies of the immune repertoire in DN have been conducted.

Through the combined analysis of high-throughput sequencing data of DN tissue and single-cell transcriptome data, this work aims to expose the pathogenic process of the immune repertoire and key stromal cells in the kidney during the course of DN. The occurrence and progression of DN is driven by alterations in gene expression regulatory factors and transcription factors. This work has provided insight into possible mechanisms behind the formation and progression of DN, which has significant implications for the development of tailored treatments for this condition.

## Method

### Data sources

The GEO database GSE142025 (10) cohort comprises high-throughput RNA-seq data (GPL20301) for 9 normal kidney tissues, 6 early DNs, and 22 late DNs. The human kidney single-cell sequencing data was collected from https://www.kidneycellatlas.org/ (12) and contains data on the transcriptome sequencing of 10928 cells. The single-cell sequencing dataset was annotated using Azimuth (https://azimuth.hubmapconsortium.org), resulting in the identification of 27 prevalent cell types in kidney tissue (**Table S1**: scRNA_annotation). The canonical signatures (canonical marker=1) of plasma cell-related cells (Plasma cell, Plasmacytoid dendritic cell) were obtained from PanglaoDB (13) (**Table S1**: Panglao_DB Plasma celltypes).

### Immune repertoire analysis

TRUST4 (14) can compute immunological repertoire data in bulk-RNA-seq samples using high-throughput RNA-seq data. We define TCR clonotypes and BCR clonotypes using sequences specific to the CDR3 (Complementary Determining Region 3) region of the variable region. The CDR3 region is a major source of diversity in the immune repertoire and plays a major role in antigen recognition (15). By using RNAseq data from the GSE142025 cohort, we predicted the immunological repertoire (TCR and BCR) of 36 samples in this work. Utilizing Immunarch (16), samples were analyzed for clonotype, immune repertoire overlap, diversity, and Gene Usage. First, sra-toolkit (17) was used to get the raw data of GSE142025 from the GEO database, then fastq-dump was utilized to convert the sra file to a fastq.gz file, and lastly, TRUST4 was utilized to infer the immunological repertoire of the data.

### Transcription factor analysis

Using master regulator analysis, the master regulatory transcription factors of normal kidney to early DN and early DN to late DN were analyzed. Employ the msViper implementation of the viper (18) R package. First, ARACNE-AP (19) was used to compute the regulatory networks of 1785 transcription factors in 36 samples, and then the msviper function was used to evaluate the activity of these transcription factors. Stringr (20) was used to build the Protein-Protein-Interaction Network, whereas Metascape (21) was utilized for functional enrichment analysis.

### Tissue microenvironment abundance analysis

CIBERSORTX (22) was used to deconvolve transcriptome data from bulk-RNAseq to understand tissue microenvironmental changes occurring throughout DN kidney tissue. To enhance the precision of deconvolution, we eliminated less abundant cell populations and combined the MNPs (mononuclear phagocytes) of four cell populations, resulting in 17 cell populations (**Table S1**). For the examination of single-cell sequencing data, Seurat4.1 (23) was used to examine single-cell sequencing data. We selected 3000 highly variable genes and downsampled 5000 cells to create the Signature Matrix. The S-mode was used to remove batch effects between single-cell and bulk-RNAseq sequencing data. Using the Impute Cell Fraction module of CIBERSORTX, the absolute scores of 17 cells from 36 samples were analyzed.

### Tissue stemness analysis

A greater number of tissue stem cells indicates a greater capability for tissue regeneration. Using stem cell taxa from the PCBC (24) database and their differentiated ectodermal, mesodermal, and endodermal progenitor cells as a training set, we employed OCLR (One Class Logistic Regression) (25) to determine the mRNAsi (mRNA stem index) of 36 tissue samples. Each stemness index was normalized to a range of 0 to 1, reflecting low to high tissue stemness.

### Pseudo-time analysis

The incidence and development of DN is a continuous process. Kidney tissue will undergo a series of organic changes from normal to early DN and finally to late DN. In order to explore the details of transcriptome changes in the occurrence and development of DN, we used monocle3 (26) to construct a pseudo-time-series change trajectory of DN according to the continuous change process of DN from control, early DN, and late DN, and obtained the simulated DN after dimensionality reduction. The result of Pseudo-time analysis use UMAP1 and UMAP2 two-dimensional scatter plot. Pseudotime represents the evolutionary trajectory of the sample.

### Weighted gene co-expression network analysis (WGCNA)

WGCNA (27) (Weighted Gene Coexpression Network Analysis) may assess the link between modules with co-expression characteristics and clinical traits based on transcriptome data. Since WGCNA is more sensitive to genes with low expression levels, genes with standard deviations below 0.5 were eliminated first. Separate WGCNA analyses were conducted. The first WGCNA employed all 36 data, established a soft threshold of seven, merged modules with feature correlations more significant than 0.80, and ultimately generated seven co-expression modules. The second WGCNA used expression data covering from control through early DN, chose a soft threshold of 4, merged modules with a feature correlation better than 0.80, and ultimately produced 13 modules.

Finally, Benjamini-Hochberg FDR correction was conducted on the Pearson correlation coefficients of the main components of these modules with Pseudotime, and Stemness Index and LogBCR_Chao1, LogTCR_Chao1. LogBCR_Chao1 and LogTCR_Chao1 are the log-transformed BCR and TCR’s Chao1. Log-transformation can make the Chao1 index fit the normal distribution. The WGCNA analysis data are included in the **Supplemental Material**. ClusterprofileR (28) was used to analyze gene lists for functional enrichment. MSIGDB (29) GO (Gene Ontology) TERM gene sets was mostly used in bulk RNA level and single-cell level. As the module’s hub genes, we first choose the genes whose absolute correlation between the genes in each module and the module’s main components is larger than 0.70. Then, SYMBOL was embedded on ENTREZID using the bitr function, followed by a study of gene enrichment using enrichGO (28). Each module’s enriched signaling pathways display the top five (Ranked By FDR).

### Statistical analysis

Using the R package psych, calculate the correlation analysis, and use corrplot to draw the correlation heat map. The R package ComplexHeatmap was utilized to draw heatmaps of differentially expressed genes, differential signaling pathway activity, and differential transcription factor activity. The ssGSEA algorithm from the R package GSVA (30) was used to evaluate the scores of gene sets in samples and cells. Wilcoxon Test was used for comparison between two groups, Kruskal-Wallis Test was used for comparison between multiple groups, and Spearman correlation coefficient was used for correlation analysis. Significance was defined as P < 0.01 (****P<0.0001, ***P < 0.001, **P < 0.01, *P < 0.05).

## Result

### Immune repertoire characteristics of DN

**Figure 1:**
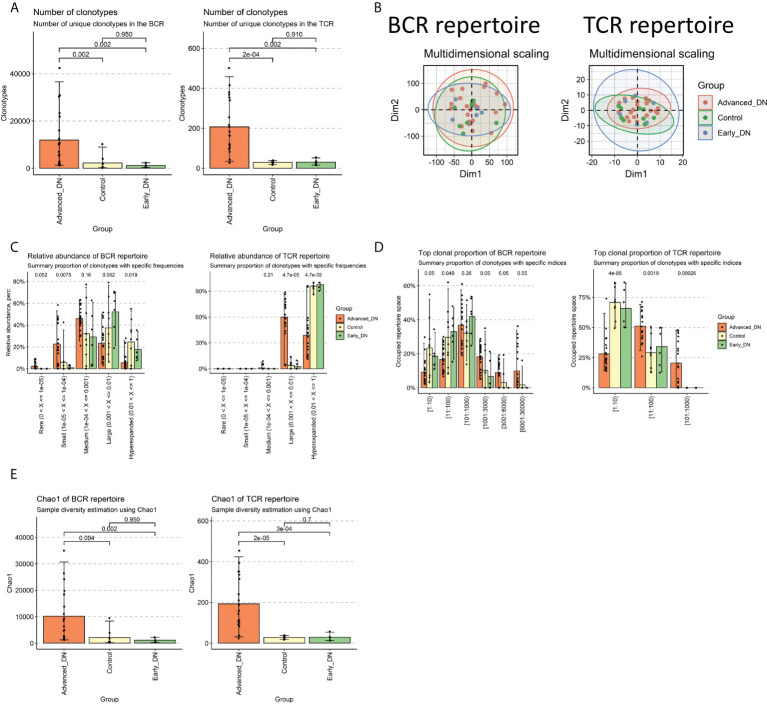
Characteristics of DN immune repertoire (BCR repertoire and TCR repertoire). **(A)** Comparison of clonotype numbers for BCR repertoire and TCR repertoire. The clonotypes here are defined according to the nucleotide sequence of CDR3. Each CDR3 sequence defines a unique clonal population. **(B)** Immune repertoire overlapping clustering. Clonal contigs of BCR repertoire and TCR repertoire were clustered separately. The number of public clonotypes is used to calculate the overlap similarity between samples (.method=“public” of the immunarch repOverlap function) **(C)** Clonal Space Homeostasis comparison. Clonal Space Homeostasis analyse relative abundance (also known as clonal space homeostasis), which is defined as the proportion of repertoire occupied by clonal groups with specific abundances. **(D)** Top clonal proportion of specific segmentation length. **(E)** Difference comparison of the clonotype diversity indicator Chao1. Bar average, error bar standard error. P values were obtained by Wilcoxon Test.

The first step was to compare the clonotype of the immune repertoires of the Control, early DN, and late DN. We found no significant difference in the number of clonotypes between healthy kidney tissue and early DN (Control vs. Early DN p>0.1, Wilcoxon Test; BCR repertoire on the left, TCR repertoire on the right), but the number of clonotypes in advanced DN was significantly different from those in early and Control (**Figures 1A**, **S1A**; Advanced DN vs. Control p<0.05; Advanced DN vs. Early DN p<0.05; BCR repertoire on the left, TCR repertoire on the right). Repertoire overlap is the most commonly used method to measure Repertoire similarity. MDS clustering of samples based on repertoire overlap revealed no significant difference between the three groups, whether BCR repertoire or TCR repertoire (**Figure 1B**). Next, the clonotypes of the three groups were examined in terms of their frequency differences (Rare:0~10^-5^, Small:10^-5^~10^-4^, Medium:10^-4^~0.001, Large: 0.001~0.01, Hyperexpanded: 0.01~1). We first performed an analysis of the BCR repertoire. Clonotype distributions at Rare and Small frequencies were found to be greater in late DN (p=0.052), whereas distributions at Large and Hyperexpanded frequencies were found to be higher in Control and early DN (p<0.05). This data suggested that the clonotype diversity of Control and early DN was low, but that of late DN was high (**Figures 1C**, **S1B**). The analysis results in the TCR repertoire show that the abundance of Large frequencies is significantly higher than that of the Early DN and Control groups, while the abundance of Hyperexpanded frequencies is significantly lower than the other two groups. Comparing the percentage difference in the number of distinct clones between the three groups revealed that the clonal population of [1:10], [11:100] was lower than that of late DN (p<0.05, Wilcoxon-Test), whereas the clonal population of [1001:3000], [3001:6000], and [6001:30,000] was higher than that of late DN (p=0.05, Wilcoxon-Test). This data revealed that the high-copy clonal population included a greater fraction of the advanced DN BCR repertoire (**Figure 1D**, **S1C**). Again we make the same comparison in the TCR repertoire. The results show that the ratio of [1:10] of Advanced DN is lower than that of Control and Early DN, while the ratio of [1:100] and [101:1000] is opposite. By using the Chao1 indicator to examine the clonal diversity of the three groups, it was determined that the Chao1 of the late DN group was considerably greater than that of the Control and early DN groups (p<0.01, Wilcoxon-Test). There was no substantial difference between Control and early DN (**Figure 1E**, BCR repertoire on the left, TCR repertoire on the right).

**Figure 2:**
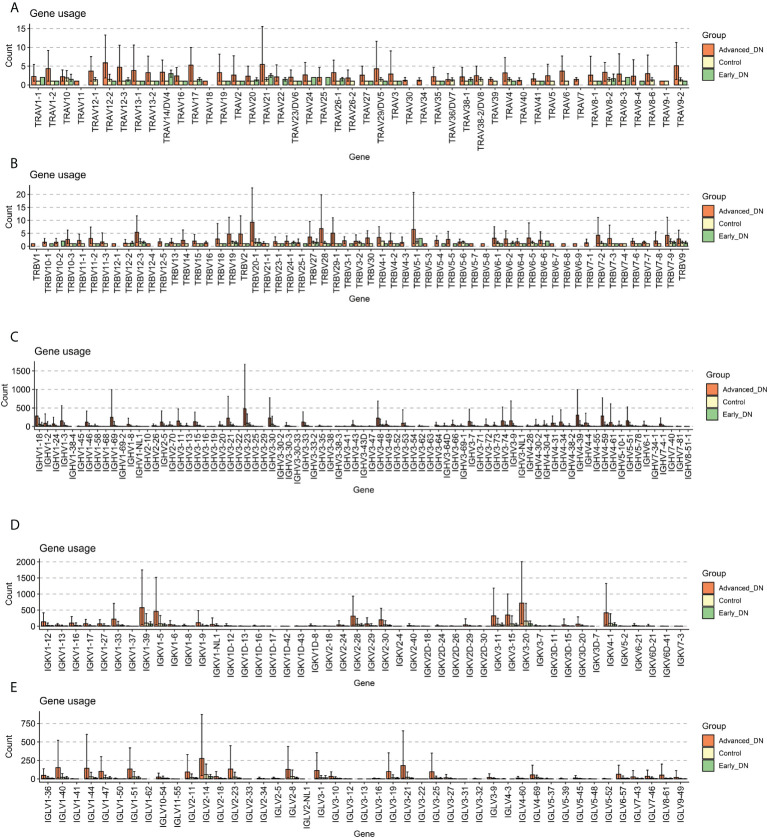
Gene usage analysis. **(A)** Comparison of Gene Usage of TRAV. **(B)** Comparison of Gene Usage of TRBV. **(C)** Gene Usage comparison of IGHV. **(D)** Gene Usage comparison of IGKV. **(E)** Comparison of Gene Usage of IGLV.

The diversity of the immune repertoire is brought about by V(D)J rearrangements, each rearranged region being expressed by a specific gene. GeneUsage refers to the V gene utilization for the transcripts. First, the geneUsages of TRAV (**Figure 2A**) and TRBV (**Figure 2B**) of the three groups were compared. The findings indicated that the number of TRAV and TRBV genes was nearly entirely less than 10; the number of late DN in different kinds of TCR genes was more than that of the other two groups. The geneUsages of IGHV (**Figure 2C**), IGKV (**Figure 2D**), and IGLV (**Figure 2E**) of the three groups, were then compared. The findings indicated that the number of IGHV, IGKV, and IGLV genes was greater than the number of TRAV and TRBV genes. IGHV3-23 genes comprised the greatest proportion of late DN IGHV genes, averaging close to 500. In late DN, the geneUsage of IGKV1-39, IGKV1-5, IGKV3-20, and IGKV4-1 was notably high. No IGLV genes come close to or surpass 500. These data show that IGKV is the immunoglobulin with the highest geneUsage in advanced DN; the expression of IGHV3-23, IGKV1-39, IGKV1-5, IGKV3-20, and IGKV4-1 may reflect the immunological response of advanced DN. Moreover, geneUsage due to immunoglobulins from B cells was substantially greater than geneUsage due to TCRs from T cells. Based on the aforementioned findings, we determined that the abundance and diversity of the immune repertoire in advanced DN were significantly greater than those in Control and early DN, whereas there was no significant difference between Control and early DN.

### Regulation of transcription factors during development of DN

**Figure 3:**
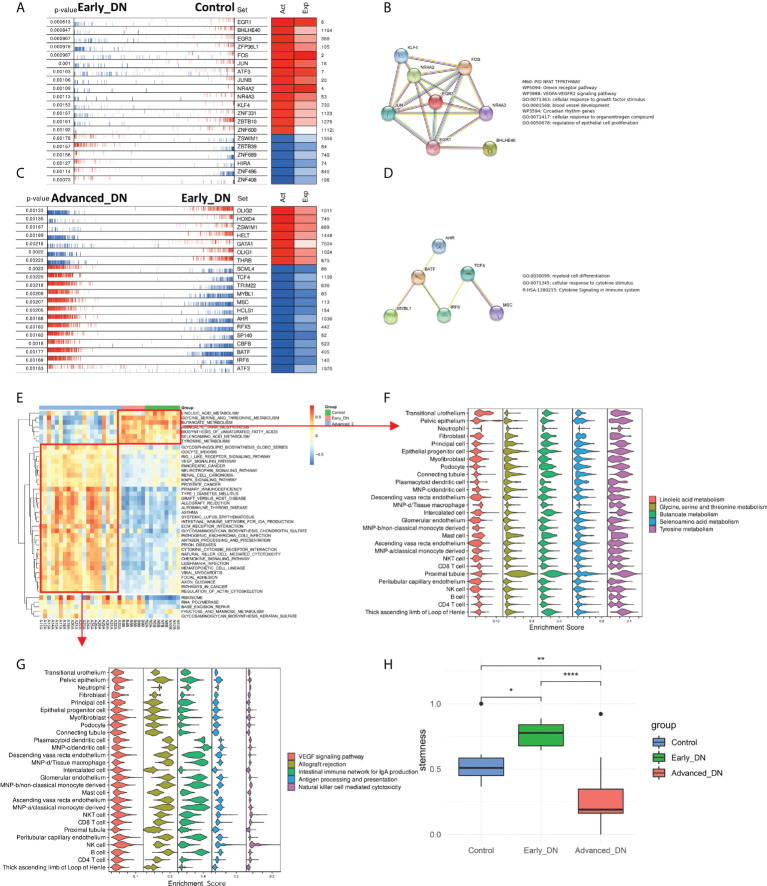
Analysis of master regulators in the occurrence and development of DN. **(A)** Analysis of control to Early DN master regulators. **(B)** The results of PPI regulatory network and functional enrichment analysis of the master regulators from Control to Early DN. **(C)** Analysis of master regulators from Early DN to Advanced DN. **(D)** The PPI regulatory network and functional enrichment analysis results of master regulators from Early DN to Advanced DN. **(E)** Results of GSVA enrichment analysis in three grouped DN samples of the MSIGDB C2 KEGG gene set. **(F)** Localization of signaling pathways enriched in Control and Early DN samples at the single-cell level. **(G)** Localization of signaling pathways enriched in Advanced DN samples at the single-cell level. **(H)** Stemness Comparison. ***P < 0.001, **P < 0.01, *P < 0.05.

Normal to early DN and early DN to late DN are the two major phases of the development of DN. Utilizing the msViper algorithm, the Master Regulators (MRs) responsible for the incidence and development of DN were analyzed. During DN emergence, we discovered that EGR1, BHLHE40, EGR3, ZFP36L1, FOS, JUN, JUNB, NR4A2, NR4A3, KLF4, ZNF331, ZBTB10, and ZNF600 were the principal transcription factors that inhibited DN occurrence, whereas ZSWIM1, ZBTB39, ZNF689, HIRA, ZNF496 and ZNF408 are the principal regulators that promote DN (**Figure 3A**). By constructing a PPI network of major transcription factors that inhibit the onset of DN by Stringr and functionally annotating the network using metascape, we discovered that these transcription factors are predominantly involved in the NFAT TFPATHWAY, Orexin receptor pathway, VEGFA-VEGFR2 signaling pathway, cellular response to growth factor stimulus, blood vessel development, and other signaling pathways (**Figure 3B**). OLIG2, HOXD4, ZSWIM1, HELT, GATA1, OLIG1, and THRB hindered DN advancement, whereas SCML4, TCF4, TRIM22, MYBL1, MSC, HCLS1, AHR, RFX5, SP140, CBFB, IRF8, and ATF3 promoted DN progression (**Figure 3C**). We developed a PPI network of transcription factors that support DN progression and discovered that the majority of these transcription factors are engaged in the Myeloid cell differentiation signaling pathway (**Figure 3D**). In addition, the msViper algorithm was utilized to evaluate transcription factor activity in 36 samples; this result is comparable to that of msViper based on transcription factor heatmap enrichment (**Figure S2A**). The 36 samples were evaluated using ssGSEA to determine the signaling pathway scores of the KEGG gene sets (MSIGDB C2 KEGG). The R package limma (31) was used to do a comparative analysis of the gene set enrichment scores for the three subgroups (**Figure 3E**). The signaling pathways RIBOSOME, RNA POLYMERASE, BASE EXCISION REPAIR, and FRUTOSE AND MANNOSE METABOLISM are active in Early DN and Advanced DN, as indicated by the heatmap of the differential enrichment analysis. At the single-cell level, these signaling pathways were not enriched in a particular cell population (**Figure S2B**); Metabolism-related signaling pathways, such as LINOLEIC ACID METABOLISM and GLYCNE SERINE AND THREONINE METABOLISM/BUTANOATE METABOLISM, were activated in the Control and Early DN groups, but significantly inhibited in the Advanced DN group. LINOLEIC ACID METABOLISM was mainly enriched in Transitional urothelium at the single cell level, whereas GLYCNE SERINE AND THERONINE METABOLISM, BUTANOATE METABOLISM, SELENOAMINO ACID METABOLISM, and TYROSINE METABOLISM were primarily enriched in Proximal tubule (**Figure 3F**). Inflammation-related signaling pathways were highly active in the Advanced DN group, including ANTIGEN PROCESSING AND PRESENTATION, INTESTINAL IMMUNE NETWORK FOR IGA PRODUCTION, ALLOGRAFT REJECTION, and others. We found that INTESTINAL IMMUNE NETWORK FOR IGA PRODUCTION and ALLOGRAFT REJECTION were enriched in Plasmacytoid dendritic cell, MNP-c/dendritic cell, Descending vasa recta endothelium, MNP-d/Tissue macrophage, Glomerular endothelium, MNP-b/non-classical monocyte-derived, Peritubular capillary endothelium, B cell (**Figure 3G**). It indicates that the majority of endothelial cells and antigen-presenting cells in advanced DN are involved in signaling pathways associated with inflammation. Different states of kidney tissue are reflected by the physiological features of these signaling pathways. Finally, the RNA Stemness Index of the three sample groups was analyzed (**Figure 3H**). Early DN stemness was substantially greater than that of the Control (p<0.05, Wilcoxon-Test) and late DN group (p<0.001, Wilcoxon-Test), although late DN stemness was significantly lower than that of the Control (p<0.01, Wilcoxon-Test). This finding suggests that in the early stages of DN, the kidney is significantly drier than in the later stages.

### Changes in the microenvironment of tissues during the onset and progression of DN

**Figure 4:**
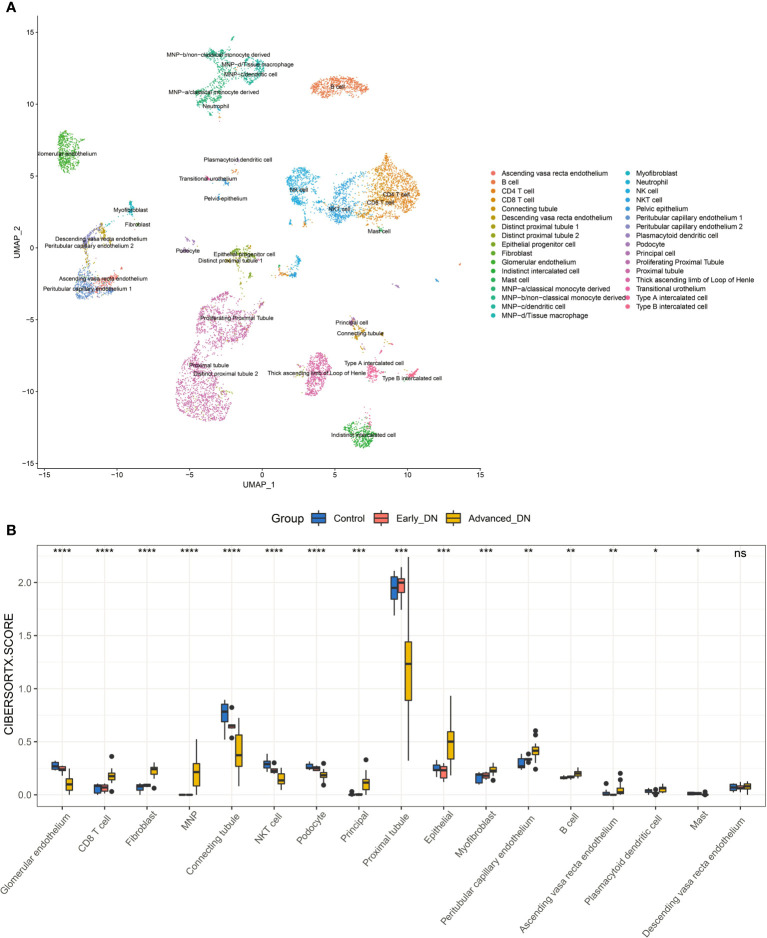
Kidney tissue microenvironment in patients with diabetic nephropathy. **(A)** Dimensionality-reduced clustering plot of reference kidney single-cell sequencing data. **(B)** Comparison of microenvironmental components in DN kidney tissue. Wilcoxon-Test was used for statistical testing. ****P<0.0001, ***P < 0.001, **P < 0.01, *P < 0.05.

Alteration in the different cellular components of the microenvironment of renal tissue might represent the principal pathophysiological processes behind the development and progression of DN. Using data from single-cell sequencing to generate a feature matrix with CIBERSORTX, we achieved absolute scores for 17 cell types across 36 samples (**Figures 4A, B**). Despite the Descending vasa recta endothelium, the other 16 cells exhibited significant variations across the three sample groups (Kruskal-Wallis Test). In late DN, scores for CD8 T cell, MNP, Peritubular capillary endothelium, Plasmacytoid dendritic cell, Epithelial, Principal, and Fibroblast were significantly higher than in the other two groups, whereas scores for Proximal tubule, Connecting tubule, Glomerular endothelium, and Podocyte were significantly lower. In late-stage DN, the data reveal significant tissue inflammation, fibrosis, and stromal tissue destruction. In addition, we discovered that the proportion of NKT cells, Connecting tubule in early DN was much more than in the other two groups. It indicates that NKT cells and connecting tubules may safeguard renal function in early DN.

### Pseudo-time analysis of DN occurrence and development

**Figure 5:**
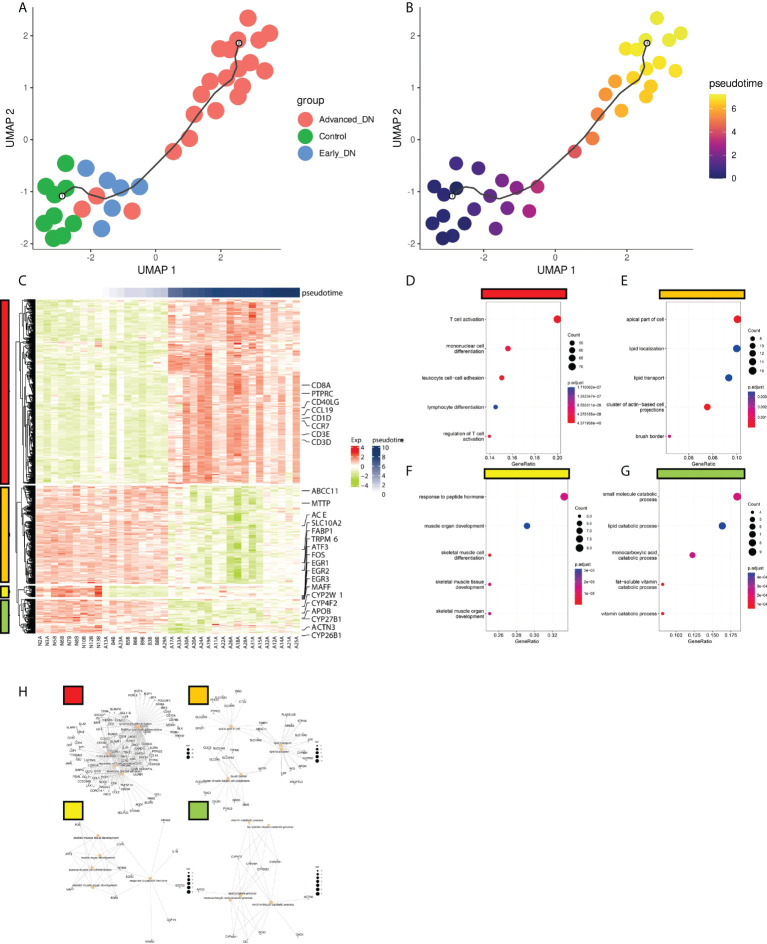
Pseudo-time analysis of DN occurrence and development. **(A)** Distribution of DN samples on the quasi-series plot. **(B)** Disease progression in DN patients deepens with Pseudotime curve. **(C)** Gene expression modules driving DN progression. There were four modules here (red, orange, yellow, green). **(D,E,F,G)** The functional enrichment analysis results of the four gene expression modules (KEGG genesets). **(H)** The gene enrichment network information map of the four gene expression modules.

Monocle3 was used to develop a pseudo-time series model of DN based on the incidence and development process of DN, utilizing transcriptome data from 36 samples. The map reveals that the spatial distance between the Control group and Early DN is short, but the gap between Early DN and Late DN is longer. In addition, the expression profile data of three late DN were grouped with those of early DN, possibly due to individual variances in DN diagnostic markers (**Figure 5A**). We defined the Control group as the starting point of Pseudo-time, and we found that the occurrence and development of DN was an approximately straight line on the UMAP 2D scatterplot (**Figure 5B**). This result indicates that the process of the occurrence and development of DN is continuous in space and time. Using the graph_test function, 731 genes with significant spatial correlation with Pseudo-time (neighbor_graph = “principal_graph”, q.value<0.001) were found. We grouped these genes into four categories using a hierarchical clustering technique (**Figure 5C**). Cluster1 is positively correlated with Pseudotime, including the genes CD3E, CD3D, CD40LG, CCR7, CD27, CD1D, CCL19, PTPRC, and CD8A, which are primarily enriched in T cell activation, mono-nuclear cell differentiation, leukocyte cell-cell adhesion, lymphocyte differentiation, and signaling pathways such as regulation of T cell activation (**Figures 5D, H**). We clustered these genes with a hierarchical clustering algorithm to obtain 4 clusters (**Figure 5C**). Among them, cluster1 is positively correlated with Pseudo-time, including CD3E, CD3D, CD40LG, CCR7, CD27, CD1D, CCL19, PTPRC, CD8A and other genes, mainly enriched in T cell activation, mononuclear cell differentiation, leukocyte cell-cell adhesion, lymphocyte differentiation, regulation of T cell activation (**Figures 5D, H**). Cluster2 is inversely correlated with Pseudo-time, containing FABP1, ABCC11, ACE, MTTP, SLC10A2, ABCC11, TRPM6 and other genes, which are primarily enriched in the apical portion of the cell, lipid localization, lipid transport, cluster of actin-based cell projections, and brush border, among others (**Figures 5E, H**). Cluster3 is up-regulated in early DN and down-regulated in late DN, including FOS, ATF3, MAFF, EGR1, EGR2, EGR3, and other genes, which are predominantly enriched in response to peptide hormone, muscle organ development, skeletal muscle cell differentiation, skeletal muscle tissue development, and other signaling pathways (**Figures 5F, H**). Cluster4 is up-regulated in Control and terminal DN but down-regulated in early DN, including ACTN3, CYP4F2, CYP2W1, CYP26B1, CYP27B1, APOB, mainly enriched in small molecule catabolic process, lipid catabolic process, monocarboxylic acid catabolic process, fat-soluble vitamin catabolic process, vitamin catabolic process and other signaling pathways (**Figures 5G, H**).

### The association between renal tissue microenvironment, disease development, and immune repertoire diversity

**Figure 6:**
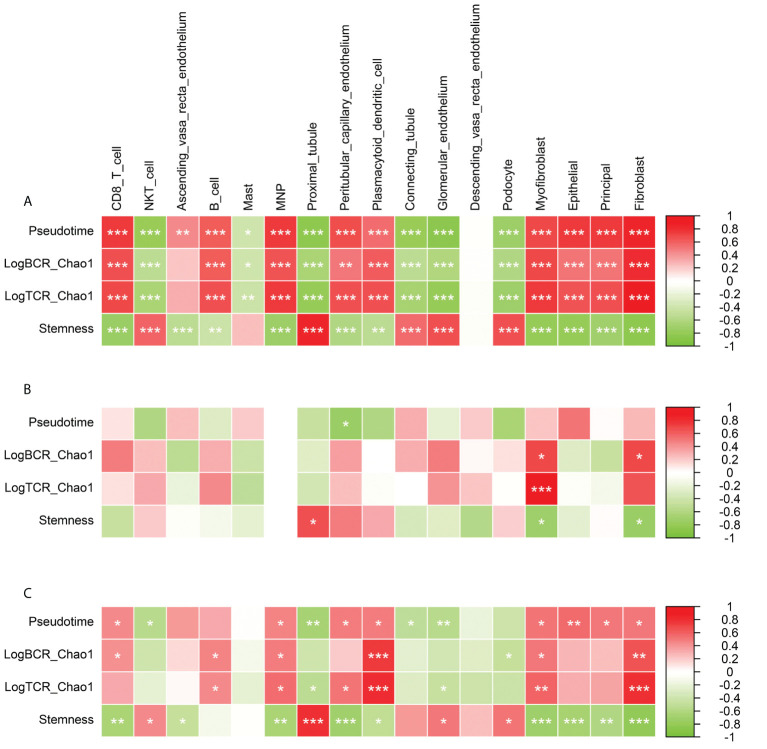
Correlation analysis of Pseudotime, LogBCR_Chao1, LogTCR_Chao1, Stemness and various cellular components in tissue microenvironment. **(A)** Correlation analysis of the scores of 17 types of cells and Pseudo time, LogBCR_Chao1, LogTCR_Chao1, Stemness during the occurrence and development of DN. **(B)** Correlation analysis of 17 cell scores and Pseudotime, LogBCR_Chao1, LogTCR_Chao1, Stemness during the early occurrence of DN. **(C)** Correlation analysis of 17 cell scores and Pseudotime, LogBCR_Chao1, LogTCR_Chao1, Stemness during the progression of DN. Spearman correlation analysis for correlation comparison. ***P < 0.001, **P < 0.01, *P < 0.05.

To explore the relationship between immune repertoire diversity, tumor stemness, Pseudotime and tissue microenvironment, we analyzed the correlation between tissue microenvironment and these indicators at different stages (Pearson). Overall, Pseudotime was significantly positively correlated with CD8 T cell, B cell, MNP, Peritubular capillary endothelium, Myofibroblast, Epithelial, Principal, Fibroblast (p<0.001), and was significantly negatively correlated with NKT cell, Proximal tubule, Connecting tubule, Glomerular endothelium, Podocyte(p<0.001) (**Figure 6A**). During the formation and progression of DN, endothelial cells convert into antigen-presenting cells and promote the activation of inflammation-related signaling pathways and the proliferation of inflammation-related cells. From normal to early stage, the LogBCR_Chao1 was mainly related to Myofibroblast and Fibroblast, while the Stemness is related to Proximal tubule and Peritubular capillary endothelium (**Figure 6B**). The correlation between Pseudotime and NKT, plasmacytoid dendritic cell, was negative. It demonstrates that immune cells enhance the progression of DN in its early stages. However, LogBCR_Chao1 was most significantly correlated with B, Plasmacytoid dendritic cell, Myofibroblast, and Fibroblast from the early to the advanced stage, which explained why the clonal population of B cells increased (**Figure 6C**). Positive correlations were found between Stemness and Proximal tubule, Glomerular endothelium, and Podocyte. Positive correlations were found between Pseudotime and Ascending vasa recta endothelium, Peritubular capillary endothelium, and Myofibroblast. The description shows that cell damage may be connected with the advancement of DN.

To explore the relationship between the immune repertoire and its immune cells, we compared the signature scores of Plasma cell and Plasmacytoid dendritic cell (Plasma_cell_Panglao, Plasmacytoid_dendritic_cell_Panglao; ssGSEA algorithm) with Pseudotime, LogBCR.Chao1, LogTCR.Chao1, Stemness, CD8 T cell, B cell, Correlation of Plasmacytoid dendritic cells (Spearman). The results showed that at the stage of DN progression, these two signature scores were significantly positively correlated with Pseudotime, LogBCR.Chao1, LogTCR.Chao1, CD8 T cell, B cell, Plasmacytoid dendritic cell, but negatively correlated with Stemness (P>0.01, Spearman). In the stage of DN occurrence, only Plasma_cell_Panglao was found to be significantly negatively correlated with Stemness (P<0.05, Spearman; **Figures S3, A–C**). This result suggests that tissue infiltration of plasma cells and secretion of immunoglobulins may play an important role in the progression of DN. In addition, we also compared the CIBERSORTX scores of CD8 T cells and B cells in three groups (Advanced_DN, Early_DN, Control). The results showed that in the process of DN, the tissue infiltration level of B cells was significantly higher than that of CD8 T cells. In the Advanced_DN group, the score of B cells was higher than that of CD8 T cells, but it was not significant (P=0.055, Wilcoxon Test; **Figure S3D**).

### The co-expression regulatory modules of DN are involved in the occurrence and development of diseases

**Figure 7:**
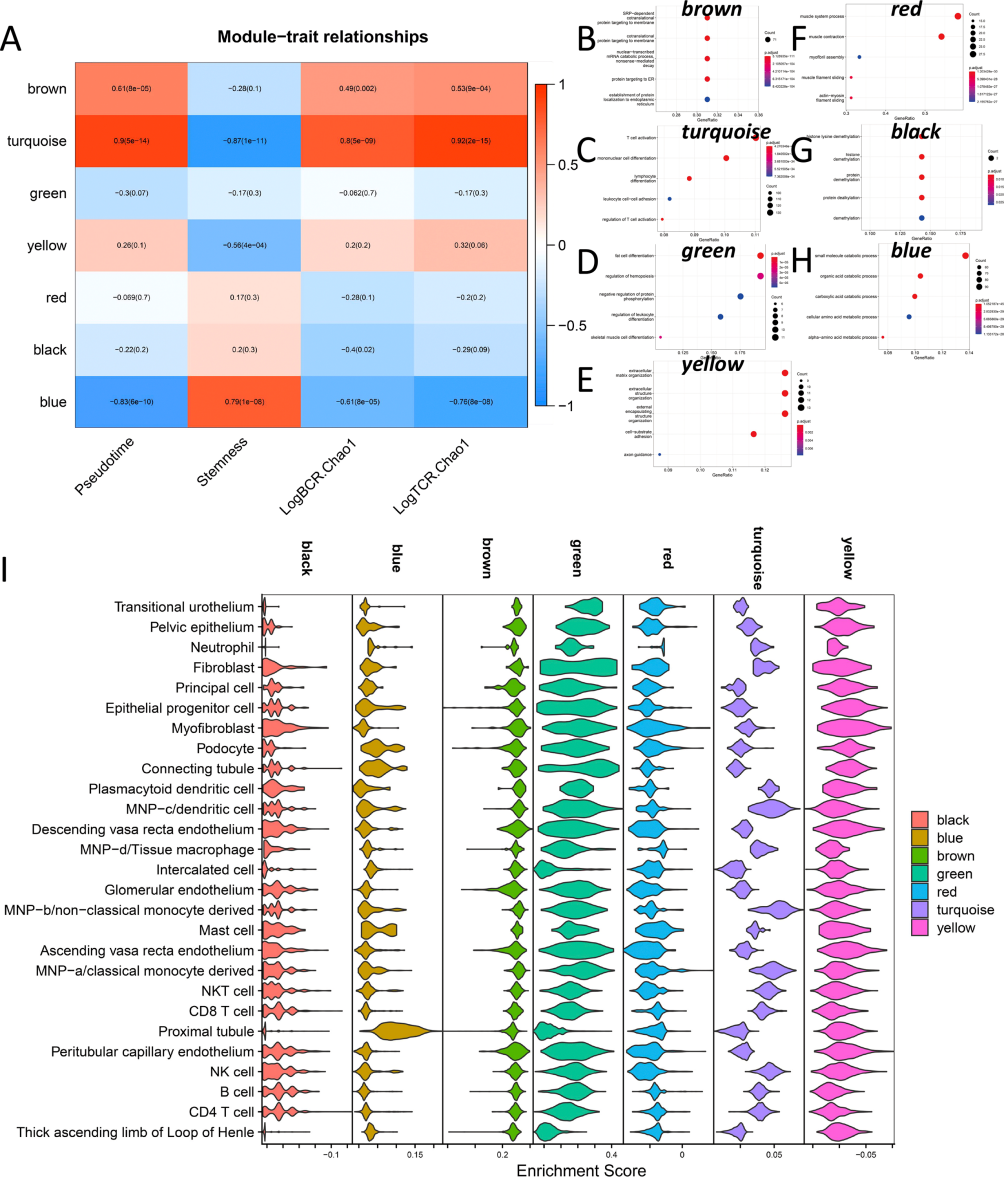
Co-expression network analysis during DN occurrence and development. **(A)** Correlation of 7 co-expression modules with Pseudotime, Stemness, LogBCR_Chao1, LogTCR_Chao1. **(B–H)** Functional enrichment analysis of seven co-expression module hub genes. **(I)** Enrichment of the hub genes in 7 co-expression modules at the single-cell level.

WGCNA was used to explore the major regulatory modules during DN progression. Finally, seven co-expression modules were obtained from the expression profile data of all 36 samples (**Figure 7A**). We performed functional enrichment analysis on the hub genes of these seven modules, respectively (**Figures 7B–H**). The blue module has the highest correlation with Stemness (R=0.79, p<0.001), while the Turquoise module has the highest correlation with LogBCR_Chao1(R=0.92, P<0.001) and LogTCR_Chao1(R=0.8, P<0.001). The Blue module is mainly involved in the small molecule catabolic process, organic acid catabolic related to the carboxylic acid catabolic process, cellular amino acid metabolic process, and alpha-amino acid metabolic process. The primary function of the turquoise module is T cell activation. Single sample enrichment analysis (ssGSEA) was done on the hub genes(abs(R)>0.7) of these modules (**Figure 7I**). The results demonstrated that the blue module was predominantly enriched in the Proximal tubule, whereas the turquoise module was predominantly concentrated in immune regulation-related cells (Fibroblast, Dendritic cell, monocyte, NK cell, CD8 T cell, CD4 T cell). This result indicates that the Proximal tubule increases stemness mostly through the blue regulatory module. The turquoise module of immune regulation-related cells is primarily responsible for DN progression and increased immune repertoire diversity. The WGCNA module clustering and phenotypic correlation data are shown in **Figure S4**.

### Cellular and molecular biological mechanisms of DN occurrence

**Figure 8:**
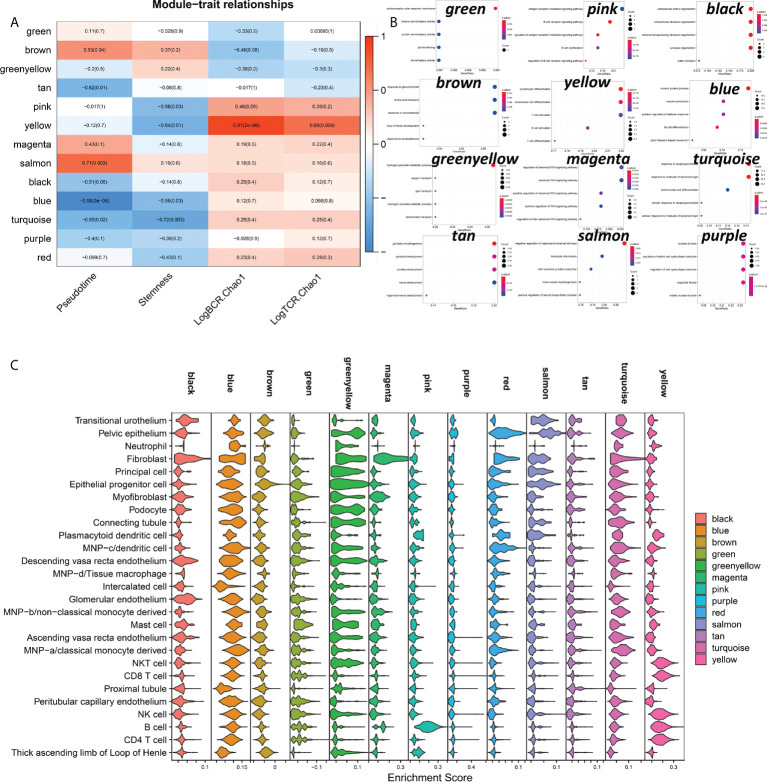
Analysis of co-expressed regulatory modules in the early stage of DN-ogenesis. **(A)** Correlation of 13 co-expression modules with Pseudotime, LogBCR_Chao1, LogTCR_Chao1 Stemness. **(B)** Functional enrichment analysis of the 12-module hub genes (KEGG gene sets). The hub genes of the Red module were not enriched for statistically significant signaling pathways. **(C)** Enrichment scores for 13 co-expression modules in 27 celltypes in kidney tissue.

From the preceding investigation, we can determine that the primary hallmarks of advanced DN are the infiltration of many immune cells and the functional impairment of stromal cells. However, from the graphs of illness development trajectories, we can find that the occurrence and development of DN have separate regulatory systems. Therefore, we undertook an in-depth examination of the process of normal kidney development to early DN by co-expression network analysis. Finally, we got 13 co-expression modules and did functional enrichment analysis on 13 modules (**Figures 8A, B**). We discovered a substantial association between the yellow module and LogBCR_Chao1 (R=0.91, P<0.001). The association between the Salmon module and Pseudotime was statistically significant (R=0.71, p<0.001). The central gene of the Yellow module is Predominantly enriched for lymphocyte activation and differentiation-related signaling pathways. The hub genes of the Salmon module are predominantly enriched in signaling pathways, including negative control of the response to an external stimulus, leukocyte chemotaxis, and cell-substrate junction assembly. Similarly, we performed a single-cell enrichment analysis (ssGSEA) on the hub genes of the 13 modules (**Figure 8C**). We found that the Yellow module was mainly enriched in NKT cells, CD8 T cells, NK cells, B cells, and CD4 T cells. The Salmon module was mainly enriched in Pelvic epithelium. In addition, we also found that the black, magenta and red modules of Fibroblast were significantly activated. The pink module was significantly activated in B cells. From this, we can think that the functional change of Pelvic epithelium is a critical factor in the occurrence of DN. LogBCR_Chao1 and LogTCR_Chao1 showed a significant, consistent trend throughout our study. However, because the clone group of the TCR repertoire is small, the number of clones is also tiny, and the geneUsage is also relatively small, so we believe that the role of the TCR repertoire in developing DN is not as significant as that of BCR repertoire. Therefore, we focus on analyzing Chao1 of the BCR repertoire in the follow-up study.

## Discussion

Diabetic nephropathy(DN) is an end-stage renal disease caused by diabetes (32). Inflammatory mechanisms contribute significantly to the development of DN (33). Inflammation is recognized to have a role in the etiology of diabetes, and individuals with long-term diabetes have considerably higher levels of inflammatory blood markers (34). However, the mechanisms underlying the onset and progression of DN and its association with inflammation remain poorly understood. We investigated the properties and molecular regulatory mechanisms of the immune repertoire (TCR, BCR) throughout the emergence and development of DN using transcriptome-sequencing data.

Immune repertoire information in DN kidney tissue can reflect changes in the diversity and abundance of T and B cell clonal populations during disease progression (15). By comparing the immune repertoires of normal, early DN, and advanced DN, we discovered that the diversity and abundance of the immune repertoire of advanced DN were greatly enhanced, but the immune repertoire of early DN did not vary significantly from that of normal kidney. The same result is achieved with either BCR repertoire or TCR repertoire. This result indicates that the erosion of solid renal tissue by immune cells mainly occurs in advanced DN. In addition, we also found that the geneUsage and abundance of the B cell clonal population were much higher than that of T cells. Kidneys of diabetic patients often exhibit a characteristic pattern of immunoglobulin G  (IgG) linear immunofluorescent staining along the glomerular and tubular basement membranes. And stronger linear IgG staining was associated with higher HR of renal death (35). Patients with diabetic nephropathy and glomerular capillary IgM and C1q deposition have poor renal prognosis, suggesting that B cell-derived IgM may be involved in diabetic kidney injury (36). Activated B cells secrete antibodies and inflammatory cytokines, form immune complexes, and together with complement, penetrate the glomerulus and attack solid kidney tissue (37). These results suggest that antibodies produced by autoreactive B lymphocytes complement the immune system as a major cause of kidney damage in advanced DN. Using transcription factor enrichment analysis, we then investigated the key regulators driving DN incidence and development. The msViper algorithm measures the transcription factor activity based on the expression of their downstream target genes. Early DN was characterized by the reduced activity of transcription factors such as EGR1, BHLHE40, FOS, JUN, and ATF3 and the increased activity of transcription factors such as ZSWIM1, ZBTB39, ZNF689, and HIRA. During the progression from early to late DN, the transcription factor activities of OLIG2, HOXD4, ZSWIM1, HELT, and GATA1 reduced, whereas the transcription factor activities of SCML4, TCF4, TRIM22, MYBL1, and AHR increased. It indicates that the incidence and progression of DN have distinct molecular regulating mechanisms. DN samples were scored by the MSIGDB C2 KEGG gene sets and localized at the single cell level. We found that early DN in which activation of GLYCINE SERINE AND THERONINE METABOLISM, BUTANOATE METABOLISM, Selenoamino acid metabolism, and Tyrosine metabolism were mainly activated in Proximal tubule cells. In contrast, immune-related signaling pathways are activated in late-stage DN, mainly in immune cells.

Further, we analyzed changes in the abundance of stromal cells and immune cells during DN occurrence and development. We found that the abundance of immune cells, fibroblasts, and epithelial cells was significantly increased in late DN, while the abundance of other stromal cells was significantly decreased. This result is consistent with typical inflammation-induced tissue damage (38). Surprisingly, early DN included the greatest population of NKT cells, connecting tubule, and glomerular endothelium. NKT cells can reduce the inflammatory response generated by a variety of autoimmunity-related conditions (39). Consequently, NKT cells may have a protective effect on renal tissue in the early stages of DN. Early DN is characterized by an abundance of glomerular endothelium (40). In contrast, a joint increase in the connecting tubule and glomerular is a typical feature of localized renal hypertension (Connecting tubule glomerular feedback in hypertension). In addition, we found that the tissue stemness score was significantly higher in early DN kidneys than in normal and advanced DN kidneys. Next, we explored the main drivers of DN occurrence and development by Pseudotime analysis. We constructed the disease progression trajectory of DN using monocle3. Through correlation analysis, we found that NKT and Plasmacytoid dendritic cells may be the main cell types that inhibit the occurrence of DN during the occurrence of DN, while Proximal tubule and Peritubular capillary endothelium may be the cell types that promote the maintenance of renal tissue stemness. In early DN, immune repertoire diversity was positively correlated with Fibroblast and Myofibroblast. During DN progression, Ascending vasa recta endothelium, Peritubular capillary endothelium, and Myofibroblast are the main cell populations that promote DN progression. The diversity of the late DN immune repertoire is mainly provided by B cells, MNP, Plasmacytoid dendritic cells, Myofibroblast, and Fibroblast. We also found that the Proximal tubule of late DN is also a cell type that promotes stemness maintenance. Proximal tubule will proliferate significantly after cell damage, and stem cell-related markers will be significantly increased (41). This result suggests that proximal tubular dedifferentiation and restoration of stemness are typical of DN.

Further, we explored the characteristics of gene co-expression regulatory modules during DN occurrence and development by WGCNA analysis. First, we performed WGCNA on all 36 samples. We found that turquoise modules (mainly enriched in immune-related signaling pathways) were mainly involved in the regulation of Pseudotime and BCR repertoire Chao1, controlling DN progression and diversity of immune repertoire abundance. The hub genes of this module are mainly enriched in immune-related cell types. The blue module (which is mainly enriched in metabolic-related signaling pathways) is mainly involved in the regulation of Stemness. The hub genes of this module are mainly enriched in Proximal tubule cells. The proximal tubular dysfunction is an early event in the pathogenesis of DN (42). Although the proximal tubule is the main site of glucose reabsorption in the glomerular filtrate, proximal tubule cells mainly rely on fatty acid oxidation to meet their high energy demands (43, 44). The Randall hypothesis or the glucose-fatty acid cycle postulates that there is oxidative competition between glucose and fatty acids (45). The renal cortex is approximately 90% proximal tubules that favor beta-oxidation but are exposed to increased glucose levels during periods of hyperglycemia (46). However, high glucose levels must activate the metabolic signaling pathways related to glycolysis and the TCA cycle (47).

To explore the onset characteristics of early DN, we performed WGCNA on kidney samples from normal and early DN. The results showed that the salmon module (which mainly regulates cell migration-related signaling pathways) regulates DN generation (Pseudotime). The hub genes of this module are mainly enriched in Pelvic epithelium. The yellow module (which mainly regulates the activation of immune cells) mainly regulates the diversity of the immune repertoire. The hub genes of this module are mainly enriched in immune cells (NKT, CD8 T, NK, B, CD4 T). We also found that multiple co-expression modules (black, magenta, red, salmon) of Fibroblast were activated. This result suggests that tissue fibrosis is still a typical feature of early DN.

Finally, we investigated the immune repertoire and the mechanism of DN onset and progression, showing the alterations and regulatory mechanisms of the DN tissue microenvironment throughout the onset and progression of the disease. This work gives insight into possible contributors, and allows further work to be undertaken to establish the role of these pathways and cell populations. The majority of this research is based on the analysis of transcriptome data, and further study is required to corroborate it.

## Data availability statement

Publicly available datasets were analyzed in this study. This data can be found here: bulkRNAseq: https://www.ncbi.nlm.nih.gov/geo/download/?acc=GSE142025&format=file; scRNAseq: https://seurat.nygenome.org/azimuth/demo_datasets/kidney_demo_stewart.rds.

## Author contributions

ZY: Methodology, Software, Validation, Formal analysis, Investigation, Writing-Original Draft, Data Curation, Supervision. YZ: Writing- Review & Editing, Formal analysis, Investigation, Methodology. NH: Software, Validation. SC: Writing- Review & Editing, Formal analysis. XW: Conceptualization, Project administration, Funding acquisition. LL: Visualization, Supervision, Methodology. All authors contributed to the article and approved the submitted version.

## Funding

This study was supported by the National Natural Science Foundation of China (No. 81970717, 82000740, and 82170845), the Key Research & Developement Program of Jiangsu Province (No.BE2022853) and Medical Science and technology development Foundation, Jiangsu Province Department of Health (No. ZD2021007).

## Conflict of interest

The authors declare that the research was conducted in the absence of any commercial or financial relationships that could be construed as a potential conflict of interest.

## Publisher’s note

All claims expressed in this article are solely those of the authors and do not necessarily represent those of their affiliated organizations, or those of the publisher, the editors and the reviewers. Any product that may be evaluated in this article, or claim that may be made by its manufacturer, is not guaranteed or endorsed by the publisher.

## Supplementary material

Supplementary Figure 1 Immune repertoire characteristics of the DN tissue microenvironment. **(A)**. The heatmap of public clonotypes shared between repertoires. **(B)** Distribution of clonotype ratios with different frequencies in DN samples. **(C)** Distribution of clonotypes in DN samples with different clone numbers.

Supplementary Figure 2
**(A)** Transcription factor activity differences in DN. **(B)** The enrichment results of activated signaling pathways in normal tissues in 27 types of cells. **(C)** Heatmap of transcription factor activity associated with Pseudotime.

Supplementary Figure 3 Clonal diversity of the immune repertoire in relation to plasma cells. ssGSEA scores of Plasma cell signatures and Plasmacytoid dendritic cell signatures collected by PanglaoDB with Pseudotime, LogBCR.Chao1, Chao1, Stemness, CD8 T cell, B cell, Plasmacytoid dendritic cell correlations (Spearman). ***P<0.001, **P<0.01, *P<0.05. **(A)** Correlation analysis of Plasma_cell_Panglao and Plasmacytoid_dendritic_cell_Panglao during DN onset and progression. **(B)** Correlation analysis of Plasma_cell_Panglao with Plasmacytoid_dendritic_cell_Panglao during early onset of DN. **(C)** Correlation analysis of Plasma_cell_Panglao with Plasmacytoid_dendritic_cell_Panglao during DN progression. **(D)** Comparison of B cell and CD8 T cell infiltration scores in three subgroups (Advanced_DN, Early_DN, Control) (Wilcoxon-Test).

Supplementary Figure 4 Weighted gene co-expression network analysis. **(A)** By WGCNA, 7 co-expression modules were obtained. **(B)** In order to make the gene expression regulatory network fit a scale-free distribution, we finally chose a soft threshold of 14. **(C)** The correlation of the 7 co-expressed module genes with Stemness. **(D)** The correlation of the 7 co-expressed module genes with Immune Chaos. **(E)** Correlation of 7 co-expressed module genes with Pseudotime. Correlations are described using the pearson correlation coefficient.
