# Supplementary figures and images for: Immune repertoire and evolutionary trajectory analysis in the development of diabetic nephropathy
Source: Front Immunol. 2022 Sep 23;13:1006137. doi: 10.3389/fimmu.2022.1006137 (PMC9537376; doi:10.3389/fimmu.2022.1006137)

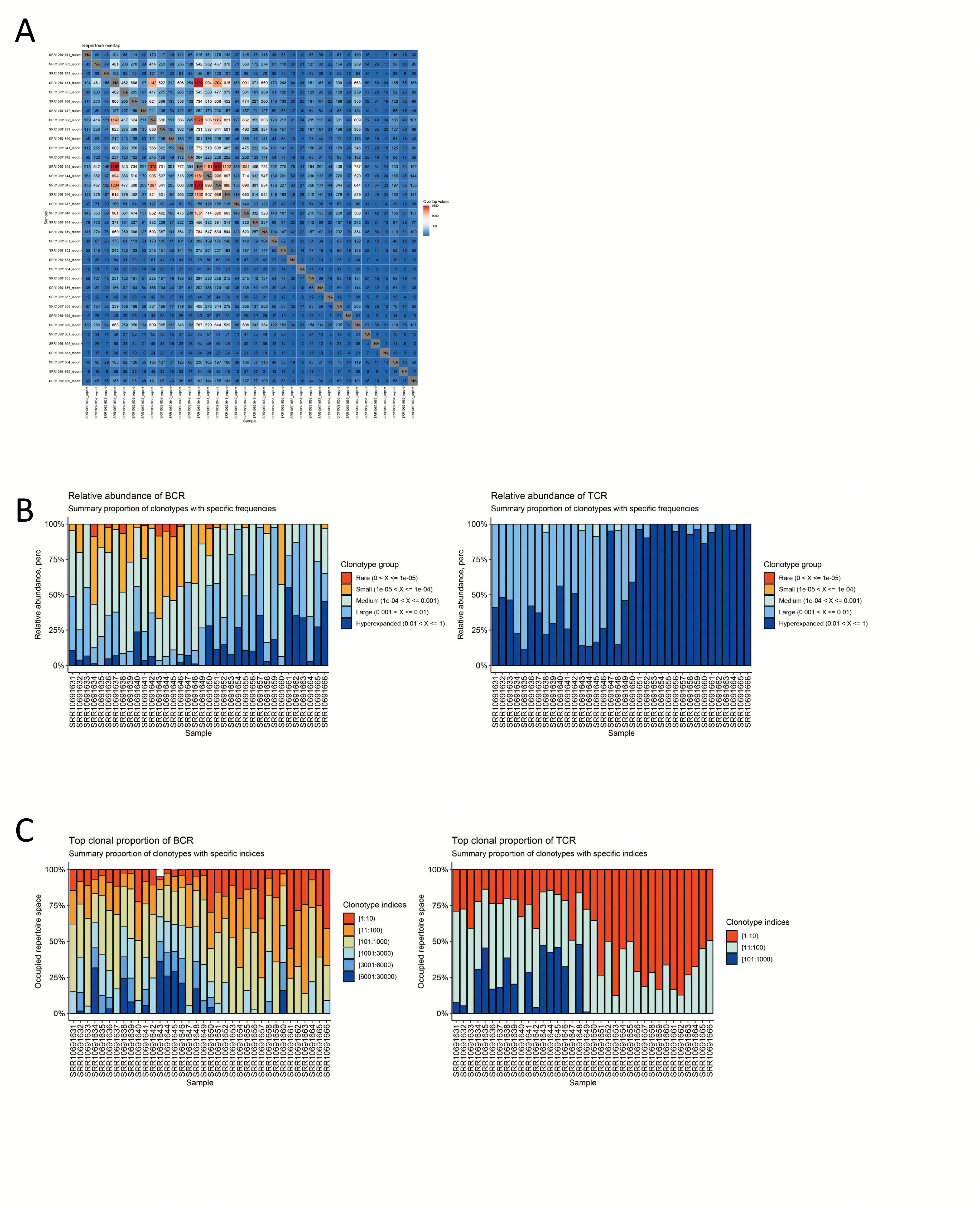

Supplement: Supplementary Figure 1 — Immune repertoire characteristics of the DN tissue microenvironment. (A). The heatmap of public clonotypes shared between repertoires. (B) Distribution of clonotype ratios with different frequencies in DN samples. (C) Distribution of clonotypes in DN samples with different clone numbers. [file Image_1.jpeg]

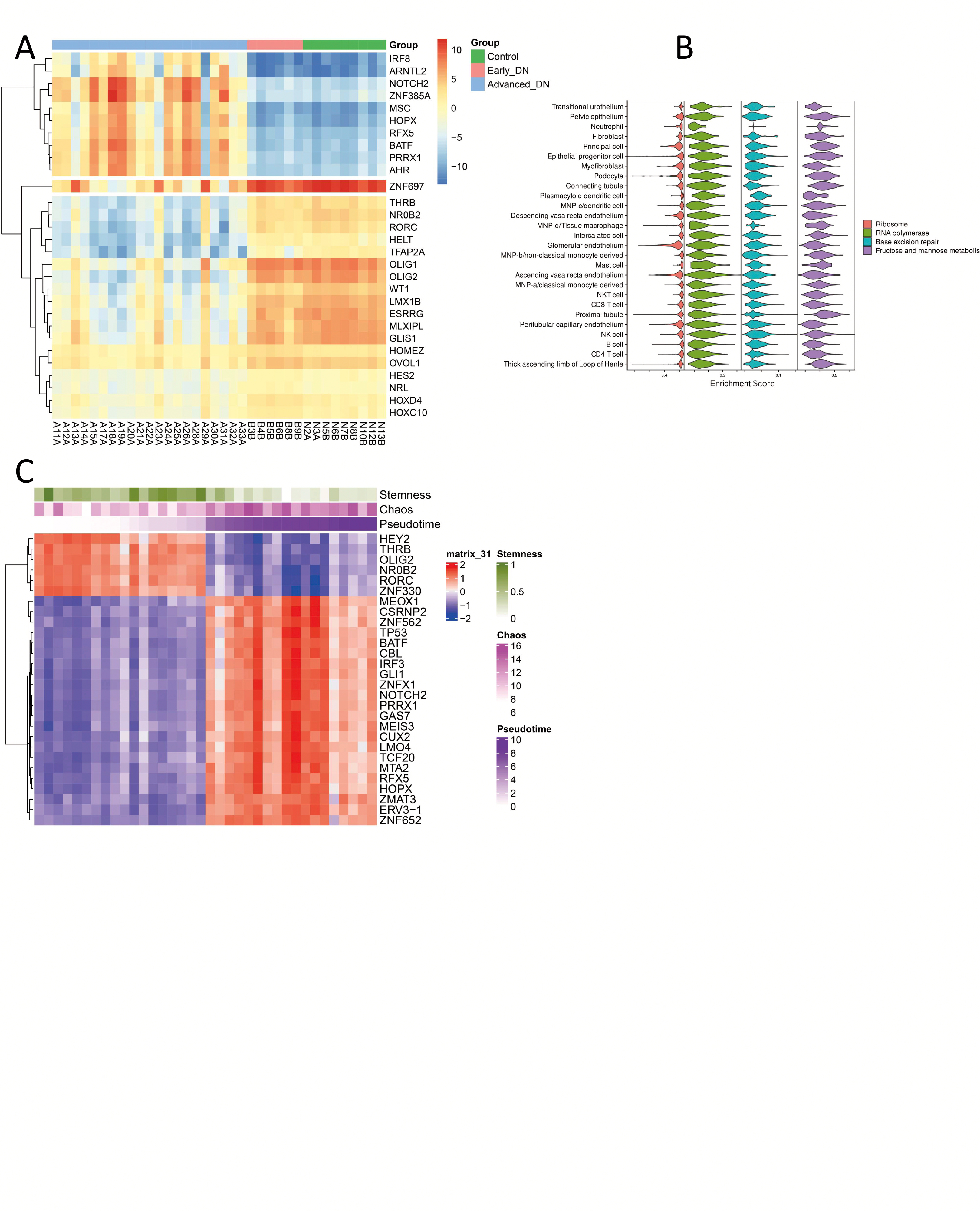

Supplement: Supplementary Figure 2 — (A) Transcription factor activity differences in DN. (B) The enrichment results of activated signaling pathways in normal tissues in 27 types of cells. (C) Heatmap of transcription factor activity associated with Pseudotime. [file Image_2.jpeg]

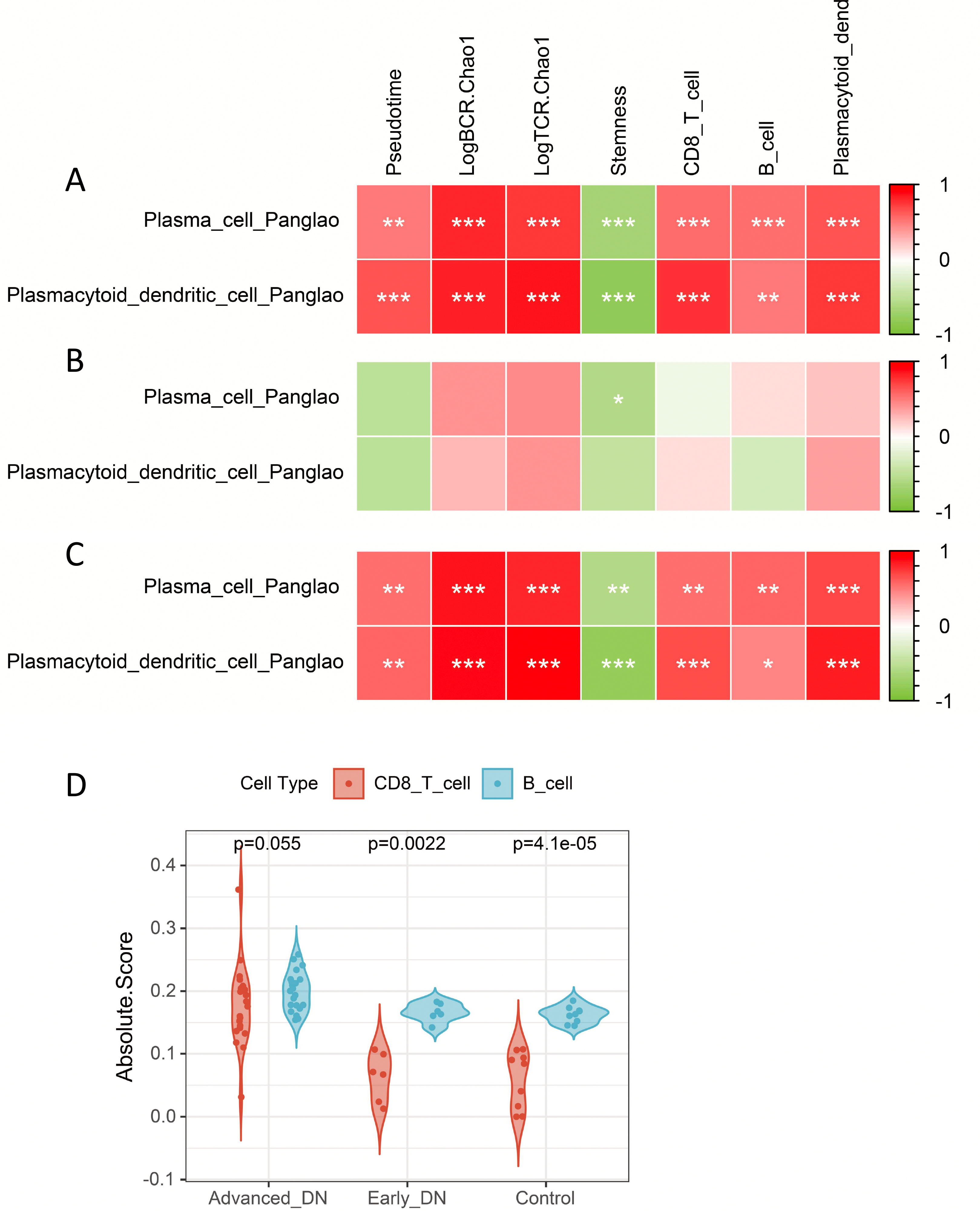

Supplement: Supplementary Figure 3 — Clonal diversity of the immune repertoire in relation to plasma cells. ssGSEA scores of Plasma cell signatures and Plasmacytoid dendritic cell signatures collected by PanglaoDB with Pseudotime, LogBCR.Chao1, Chao1, Stemness, CD8 T cell, B cell, Plasmacytoid dendritic cell correlations (Spearman). ***P<0.001, **P<0.01, *P<0.05. (A) Correlation analysis of Plasma_cell_Panglao and Plasmacytoid_dendritic_cell_Panglao during DN onset and progression. (B) Correlation analysis of Plasma_cell_Panglao with Plasmacytoid_dendritic_cell_Panglao during early onset of DN. (C) Correlation analysis of Plasma_cell_Panglao with Plasmacytoid_dendritic_cell_Panglao during DN progression. (D) Comparison of B cell and CD8 T cell infiltration scores in three subgroups (Advanced_DN, Early_DN, Control) (Wilcoxon-Test). [file Image_3.jpeg]

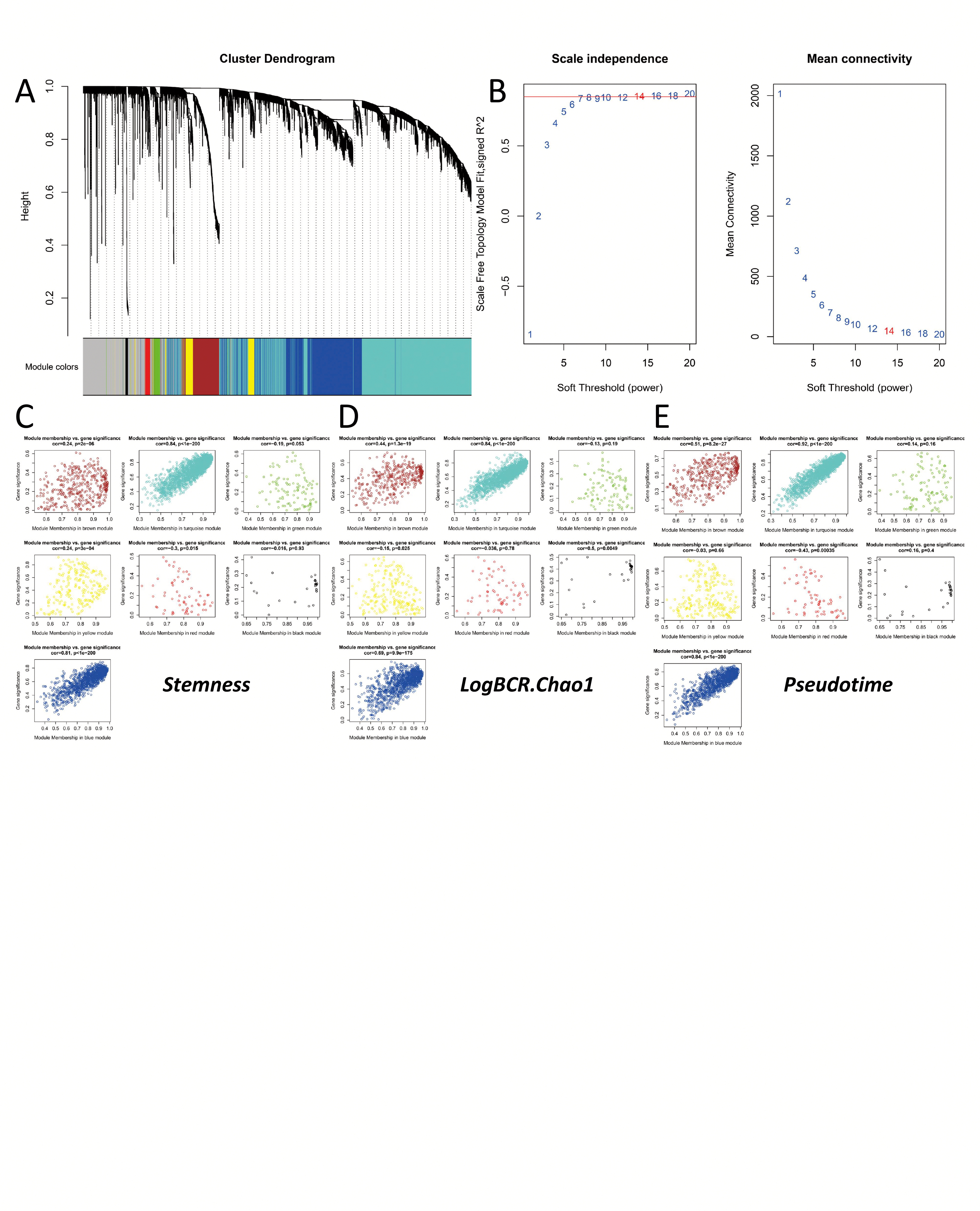

Supplement: Supplementary Figure 4 — Weighted gene co-expression network analysis. (A) By WGCNA, 7 co-expression modules were obtained. (B) In order to make the gene expression regulatory network fit a scale-free distribution, we finally chose a soft threshold of 14. (C) The correlation of the 7 co-expressed module genes with Stemness. (D) The correlation of the 7 co-expressed module genes with Immune Chaos. (E) Correlation of 7 co-expressed module genes with Pseudotime. Correlations are described using the pearson correlation coefficient. [file Image_4.jpeg]
